# Occurrence, Spatial Distribution, and Risk Assessment of PFOA and PFOS in the Henan Section of the Yellow River

**DOI:** 10.3390/toxics14060509

**Published:** 2026-06-11

**Authors:** Xianhong Sun, Yixin Liang, Lin Wang, Jingwen Wang

**Affiliations:** 1 College of Engineering, Zhengzhou Technology and Business University, Zhengzhou 451400, China; 3000013904@ztbu.edu.cn; 2 College of Geographical Sciences, Faculty of Geographical Science and Engineering, Henan University, Zhengzhou 450046, China; 3 School of Ecology and Environment, North China University of Water Resources and Electric Power, Zhengzhou 450046, China; wjw1739@163.com

**Keywords:** Yellow River Basin, per- and polyfluoroalkyl substances (PFASs), occurrence characteristics, source apportionment, ecological and human health risk assessment

## Abstract

To address the environmental evolution and management needs of emerging contaminants in the Yellow River Basin (Henan Section), China, nine typical functional cross-sections, covering industrial outfalls, sewage treatment plant (STP) effluents, human activity-dense areas, and baseline tributaries, were selected to systematically investigate the occurrence, potential sources, and multi-dimensional risks of perfluorooctanoic acid (PFOA) and perfluorooctane sulfonate (PFOS) in surface water. The results indicated a 100% detection rate of the target pollutants across all sites, with PFOA (0.45–7.46 ng/L) being the absolute dominant analogue. The spatial distribution exhibited an evident industrial point-source-driven pattern, where the pollution loads at the Jili District industrial outfall (S7) and STP effluent (S5) were significantly higher than those in non-point sources and natural baseline waters. Source apportionment suggested that direct wastewater discharge and secondary release from regional industrial clusters were likely key contributors to PFAS spatial heterogeneity. Multi-dimensional risk assessments revealed that the current ecological risk quotients (RQ < 0.01) for aquatic organisms and the human health risk values (HR < 0.1) via drinking water ingestion for various age groups were well within safe and controllable ranges. However, PFOS contributed significantly more to the ecological risk than PFOA, and children exhibited slightly higher health exposure vulnerability than adults. Although the overall risk is minimal, PFOA concentrations at high-load cross-sections have exceeded the latest stringent maximum contaminant level (4.0 ng/L) mandated by the US EPA in 2024. This study suggests an urgent need to establish a dynamic, life-cycle monitoring network for PFASs in the basin and to prioritize targeted deep-reduction strategies for high-risk industrial point sources.

## 1. Introduction

Per- and polyfluoroalkyl substances (PFASs), particularly perfluorooctanoic acid (PFOA) and perfluorooctane sulfonate (PFOS), are universally recognized as formidable emerging contaminants due to their extreme environmental recalcitrance and bioaccumulation [1,2,3]. Over recent years, global regulatory frameworks governing PFOA and PFOS have undergone an unprecedentedly stringent paradigm shift. Specifically, China’s current Standards for Drinking Water Quality (GB 5749-2022) [4] established concentration thresholds of 80 ng/L and 40 ng/L, respectively. More notably, in April 2024, the United States Environmental Protection Agency (US EPA) promulgated a rigorous national drinking water standard, restricting the Maximum Contaminant Level (MCL) for both PFOA and PFOS to 4.0 ng/L. Under these exponentially tightening spatiotemporal benchmarks, diagnosing the occurrence dynamics and health risks of these analogues in vital freshwater basins has emerged as an urgent global priority [5,6,7].

The Yellow River Basin (YRB) is a paramount ecological barrier and socio-economic corridor in China. While extensive research has investigated the occurrence of PFASs in the YRB, previous studies predominantly focused on macroscopic mainstream assessments or generalized up-mid-downstream comparisons [8,9,10]. However, this homogenized approach often masks localized contamination spikes driven by diverse riparian functions. This knowledge gap is particularly pronounced in the Henan section of the YRB. Situated in the critical geographical transition zone between the middle and lower reaches, this intensely populated region exhibits a highly complex “industrial-agricultural composite” paradigm. Consequently, the riverine environment here is highly fragmented into distinct “typical functional cross-sections,” including dense industrial wastewater outfalls, sewage treatment plant downstream zones, massive agricultural irrigation areas, and centralized drinking water sources. Each functional zone sustains divergent pollution loads and emission signatures. Relying merely on generalized mainstream monitoring intrinsically fails to capture the spatial heterogeneity of point-source emissions, nor can it objectively evaluate the exact exposure vulnerabilities of local demographics. Currently, systematic profiling that links multidimensional risks of PFOA and PFOS directly with these specific functional cross-sections remains severely underexplored.

To explicitly bridge this localized knowledge gap, this study strategically selected 9 representative functional cross-sections within the Henan section of the YRB. Utilizing ultra-performance liquid chromatography–tandem mass spectrometry, the core objectives were to: (1) characterize the precise spatial occurrence and dominant point-source attributes of PFOA and PFOS driven by divergent functional orientations; (2) map the ecological risk hotspots via a Risk Quotient model; and (3) comprehensively delineate the human health risks via drinking water ingestion for different age demographics (adults and children), strictly benchmarked against the latest rigorous international standards (e.g., the US EPA 4.0 ng/L MCL). Ultimately, this study aims to transition macro-monitoring into micro-scale source apportionment, providing a targeted scientific foundation for managing high-risk outfalls and safeguarding drinking water security in the YRB.

## 2. Materials and Methods

### 2.1. Sampling Site Selection and Sample Collection

**Figure 1:**
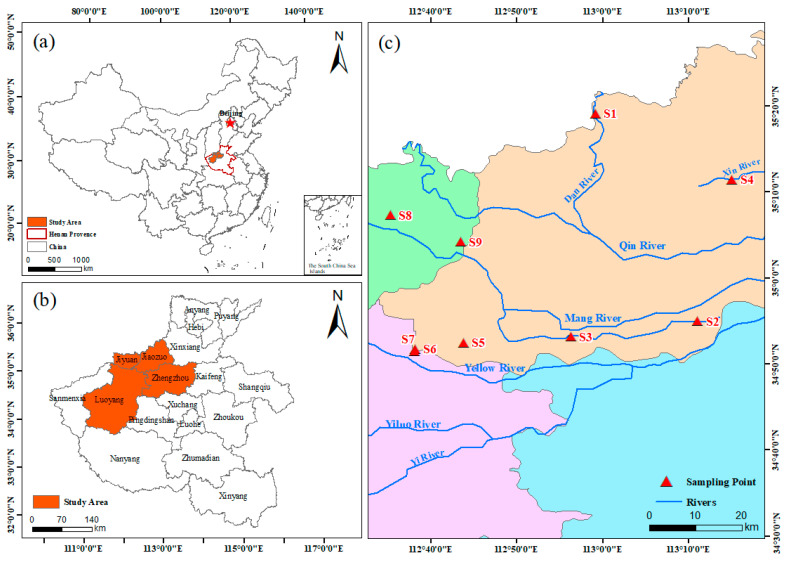
Spatial distribution of sampling sites: (**a**) China; (**b**) Hanan Provence; (**c**) Study area.

To scientifically identify source contributions, the sampling sites encompassed industrial outfall impact zones, sewage treatment plant receiving waters, densely populated human activity areas, and baseline tributaries (Table 1). A total of 9 sampling sites were established across typical functional cross-sections in the Henan section of the Yellow River Basin (Figure 1). On 30 May 2024, water samples were collected at a depth of 0.5 m below the water surface using 1 L polypropylene bottles. Prior to sampling, the polypropylene bottles were sequentially rinsed three times with ultrapure water, methanol, and the target water sample. Post-collection, the samples were hermetically sealed in the dark, refrigerated at 4 °C, transported to the laboratory within 24 h, and pretreated within 10 days.

### 2.2. Sample Pretreatment and Instrumental Analysis

#### 2.2.1. Sample Pretreatment

Native standards of the target analytes (PFOA and PFOS) and their corresponding isotopically labeled internal standards (13C4-PFOA and 13C4-PFOS) were purchased from J&K Scientific (Beijing, China). Methanol, acetonitrile, and acetic acid (HPLC grade), along with ammonium acetate (guaranteed reagent), were supplied by Thermo Fisher Scientific (Wilmington, DE, USA). Milli-Q ultrapure water (resistivity ≥ 18.2 MΩ·cm) was utilized throughout the experiments.

The sample pretreatment protocol was optimized based on the Chinese National Standard (HJ 1333-2023) [11]. Briefly, 1 L of the water sample was filtered through a 0.45 μm glass fiber filter, followed by the precise addition of an appropriate amount of internal standards to correct for matrix effects. The enrichment of target analytes was performed using an automated solid-phase extraction system (AutoEmpore, LabTech, Beijing, China) equipped with Oasis WAX SPE cartridges (6 mL, 150 mg). The operational procedure was as follows: the cartridges were sequentially conditioned with 6 mL of 0.1% methanolic ammonia, 6 mL of methanol, and 6 mL of ultrapure water. Subsequently, the water samples were loaded onto the cartridges at a constant flow rate of 5 mL/min. After loading, the cartridges were washed with an ammonium acetate buffer to eliminate impurities. Finally, the target compounds were eluted with 8 mL of 0.1% methanolic ammonia. The collected eluates were gently concentrated to 1.0 mL under a mild nitrogen stream using a nitrogen evaporator (TTL-DCI/DCII, Tongtailian, Beijing, China), filtered through a 0.22 μm organic phase syringe filter, and transferred into autosampler vials for instrumental analysis.

#### 2.2.2. Instrumental Analysis Conditions

The target pollutants were quantified utilizing an ultra-high-performance liquid chromatography-triple quadrupole tandem mass spectrometer (UHPLC-MS, Agilent 1290-6495D, Agilent Technologies, Inc., Santa Clara, CA, USA).

Chromatographic conditions: Chromatographic separation was achieved on an Agilent ZORBAX RRHD Eclipse Plus C18 column (2.1 mm × 30 mm, 1.8 μm) maintained at a constant column temperature of 45 °C, with an injection volume of 2 μL. The mobile phase consisted of (A) 2 mM aqueous ammonium acetate and (B) acetonitrile, delivered at a flow rate of 0.8 mL/min. The gradient elution program was programmed as follows: 0–1 min, maintained at 20% B; 1–2.5 min, linear increase to 50% B; 2.5–4.5 min, further increased to 90% B and held for 2 min; returning to the initial 20% B at 5.5 min to re-equilibrate the system.

Mass spectrometric conditions were as follows: the MS system was equipped with an Agilent Jet Stream electrospray ionization source, operating in negative ion mode using multiple reaction monitoring for data acquisition. The key ion source parameters were optimized as follows: capillary voltage, 2400 V; drying gas temperature, 160 °C; drying gas flow rate, 18 L/min; sheath gas temperature, 390 °C; sheath gas flow rate, 11 L/min; and nebulizer pressure, 24 psi.

### 2.3. Quality Assurance and Quality Control

To strictly prevent baseline contamination, contact with polytetrafluoroethylene or any other fluoropolymer materials was meticulously avoided throughout the entire process, encompassing sampling, storage, and pretreatment. Procedural blanks, procedural duplicates, and matrix-spiked samples were concurrently analyzed in each batch to implement quality control. The test results indicated that no target analytes were detected in any procedural blanks, effectively eliminating the potential for cross-contamination during the experiments.

Quantification was performed employing the isotope-dilution internal standard method. The target analytes exhibited excellent linearity over the established concentration gradients, with correlation coefficients (R^2^) exceeding 0.9999. The relative standard deviations (RSDs) for the duplicate samples were strictly below 10%, indicating robust analytical reproducibility and instrumental stability. The sampling campaign in this study encompassed diverse functional cross-sections within the Yellow River Basin, including river tributaries and wastewater treatment plant effluents. Notably, the collected wastewater treatment plant samples exclusively comprised compliant final effluents (i.e., treated water meeting discharge standards), which intrinsically contained relatively low levels of dissolved organic matter and co-extracted impurities. Consequently, negligible ion suppression effects were observed during the UHPLC-MS analysis. To further ensure quantitative accuracy, all matrix spike recovery tests were rigorously performed using these actual environmental water samples.

Furthermore, the matrix spike recoveries for PFOA and PFOS ranged from 86.4% to 89.7%, adequately fulfilling the requirements for trace quantification in complex surface water matrices. The method limit of quantification for both target compounds was uniformly set at 1.2 ng/L. All surface water samples were preconcentrated 1000-fold via solid-phase extraction. Accounting for this concentration factor, the effective method quantification limit for the original water samples was 0.0012 ng/L.

### 2.4. Comprehensive Risk Assessment Models

#### 2.4.1. Ecological Risk Assessment Model

The internationally widely adopted Risk Quotient (RQ) method was employed to quantitatively evaluate the potential ecological risks posed by PFOA and PFOS to the aquatic ecosystem. The RQ was calculated using the following equation [12]:

(1) $$\mathrm{RQ}=\frac{\mathrm{MEC}}{\mathrm{PNEC}}$$

where MEC represents the measured environmental concentration of the pollutant in the water (ng/L), and PNEC denotes the predicted no-effect concentration (ng/L). An RQ < 0.01 indicates a low (negligible) risk, 0.01 ≤ RQ < 1 denotes a moderate potential risk, and an RQ ≥ 1 signifies a high risk [13].

#### 2.4.2. Human Health Risk Assessment Model

Given the extreme environmental persistence and bioaccumulation of PFASs, coupled with the fact that the surface water of the Yellow River Basin serves critical water supply and agricultural irrigation functions, evaluating the exposure risks to surrounding populations holds significant early-warning value. Among various exposure routes (dietary, inhalation, and dermal contact), drinking water ingestion remains the primary pathway for human exposure to PFASs in aquatic environments [14]. Consequently, this study employed the Health Risk (HR) index as the core metric to conduct a quantitative health risk assessment via drinking water ingestion for specific demographic groups (adults and children) [15,16]. The calculation formulas are as follows:

(2) $$\mathrm{HR}=\frac{\mathrm{EDI}}{\mathrm{RfD}}$$

(3) $$\mathrm{EDI}=\frac{c\times \mathrm{IR}}{\mathrm{BW}}$$

where EDI is the estimated daily intake of pollutants via drinking water (ng/(kg·d)), c is the measured concentration of PFOA and PFOS in the water (ng/L), IR is the daily ingestion rate of drinking water (L/d), and BW is the average body weight (kg). RfD represents the reference dose of the pollutant (ng/(kg·d)), which estimates the maximum daily intake that is not anticipated to cause adverse health effects over a lifetime of exposure.

Based on the calculated HR values, health risks were categorized into three tiers: HR < 0.1 indicates no significant health risk; 0.1 ≤ HR ≤ 1 implies a potential health risk; and HR > 1 signifies a direct health risk.

## 3. Results and Discussion

### 3.1. Spatial Distribution and Source Apportionment

**Figure 2:**
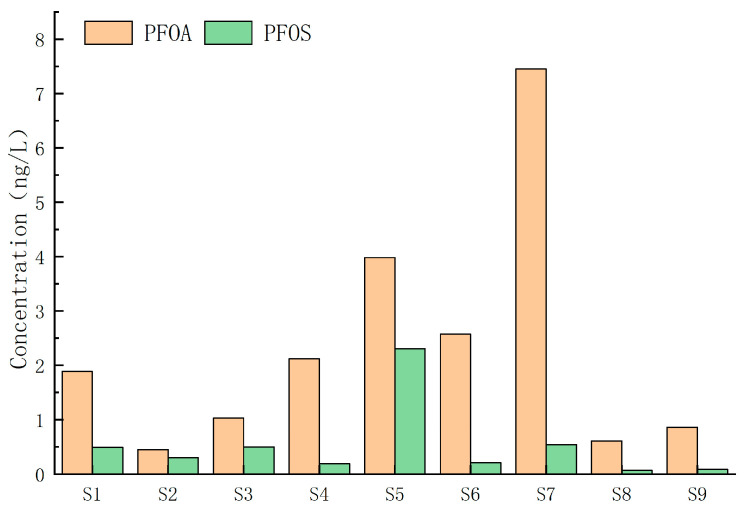
Concentrations and spatial distribution of PFOA and PFOS at different sampling sites in the surface water of the Yellow River (Henan Section).

Across the 9 monitoring cross-sections in the Henan section of the Yellow River Basin, the detection frequencies of both PFOA and PFOS reached 100%, with the mass concentrations of PFOA being significantly higher than those of PFOS at all sites. This spatial distribution and contamination profile (Figure 2) are primarily driven by surrounding land-use patterns and industrial configurations. In typical point-source impact zones, such as the receiving waters of industrial sewage treatment plants (STPs) and discharge outfalls, the pollutants exhibited a pronounced “industrial signature.” Specifically, the Jili District River outfall (S7) emerged as the most severe PFOA contamination hotspot within the entire monitoring network. The PFOA concentration at the downstream S7 cross-section was nearly three times higher than that at the upstream site (S6), indicating that this direct outfall constitutes an exceptionally strong point-source input. Concurrently, the PFOA concentrations in the effluents of the three industrial STPs displayed a decreasing gradient: S5 (Mengzhou) > S4 (Wuzhi) > S9 (Jiyuan). Among them, driven by high-fluorine-consuming industries within the Mengzhou industrial agglomeration area, such as leather manufacturing, paper production, and fine chemicals, both PFOA and PFOS levels at S5 were elevated. Conversely, cross-sections S4 and S9 primarily receive wastewater from new materials and equipment manufacturing, resulting in a pollutant composition absolutely dominated by PFOA. This phenomenon is highly consistent with the point-source contamination profiles widely reported in adjacent waters of domestic fluorochemical parks in Changshu and Taicang [17,18,19,20,21]. Based on the observed data characteristics, industrial wastewater discharge emerges as the most probable driver of chronic PFAS contamination in the basin. This finding underscores the necessity for immediate source identification and targeted regulatory interventions at identified high-pollution nodes.

Beyond intensive industrial point sources, intensive human activities constitute a non-point source input that cannot be overlooked. Taking the Qingtian River (S1) as an example, which serves dual functions as a drinking water source and a tourist area, the concentrations of PFOA (1.89 ng/L) and PFOS (0.49 ng/L) exhibited a moderate contamination level. Its pollution load most likely originates from the frequent use of fluorinated detergents and personal care products during tourist activities, coupled with the intermittent discharge of adjacent domestic sewage. This characteristic aligns well with the water contamination patterns observed in Rizhao, a typical tourist city [22], indicating that in comprehensive basin management, high vigilance is required regarding the latent risks posed by such non-point human activities to the water quality of sensitive water sources.

In contrast, the PFAS loads in traditional agricultural areas and baseline tributaries were maintained at extremely low levels. At the Panxi River (S8) cross-section, which is primarily influenced by agricultural non-point sources, the concentrations of PFOA and PFOS were diminutive, with PFOS even approaching the method limit of detection. This suggests that, in the absence of a history of specific fluorinated pesticide application, the substantive contribution of traditional agricultural cultivation to non-point source PFAS contamination is highly limited. This conclusion corroborates the findings of Zeng et al. [23] in the Pearl River Delta. Furthermore, no significant point-source inputs were identified at sections S2 and S3 of the Mang River. Consequently, governed by long-distance hydrodynamic dilution and the adsorption-sedimentation of suspended particulate matter, overall pollutant concentrations remained at relatively low levels.

### 3.2. Occurrence and Source Apportionment of PFASs

The distribution of PFASs in the surface water of the Yellow River Basin exhibits a pronounced dual nature characterized by “regional heterogeneity” and “anthropogenic-driven” attributes. A systematic integration and comparison of the empirical data from this study with historical literature across the entire basin (Table 2) reveals that the pollutant compositions and concentration gradients in different reaches are highly coupled with adjacent industrial categories and urbanization processes. Despite the global fluorochemical industry’s ongoing transition toward short-chain alternatives, the highly recalcitrant PFOA and PFOS remain the predominant contaminants in the Yellow River Basin. This is profoundly influenced by their massive historical usage baseline and environmental lag effects.

Regarding spatial evolution, the basin-wide pollution load is linked to the industrial layout along the river. In upstream tributary regions, such as the Huangshui River (Qinghai section), extraordinarily high levels of PFOA (>3000 ng/L) have been previously monitored. This is primarily attributed to the direct discharge and subsequent secondary release of high-concentration wastewater from specific local industries, including carpet manufacturing and textile dyeing. In the mid- and downstream mainstream and tributaries, the contamination profile gradually transitions into a superimposed driving pattern of urbanization and heavy industry. For instance, the water quality of the Fenhe River (Shanxi section) is predominantly impacted by the intertwining of dense heavy industrial bases and municipal domestic sewage, with its PFOA and PFOS concentrations slightly exceeding the averages reported in this study. Conversely, downstream tributaries like the Beiluo River exhibit industry-specific contamination signatures strongly driven by petroleum exploration and extraction activities. By comparison, the overall concentration in the Henan section of the Yellow River remains at a moderately low level, substantiating the significant physical dilution and natural attenuation of upstream inflows during long-distance transport. Nevertheless, the composite industrial-agricultural pollution paradigm of “fine chemicals and advanced equipment manufacturing” precisely identified in this reach provides a crucial regional supplement to the environmental behavior profile of emerging contaminants in the mid- and lower Yellow River Basin.

Through a horizontal basin-wide comparison, the input sources of PFASs in the Yellow River Basin can be clearly delineated into distinct hierarchical evolutionary patterns. First, industrial point sources constitute the key driving mechanism behind the formation of elevated localized pollution burdens. Whether originating from fluorochemical and textile processing upstream, petroleum extraction downstream, or the leather and fine chemical parks in the Henan section observed in this study, the variance in industrial categories directly dictates the fingerprint characteristics and upper concentration limits of PFASs in localized water bodies. Second, wastewater treatment plants may emerge as critical nodes for secondary emissions within the watershed. Because conventional STPs are primarily designed to abate traditional organic loads and lack targeted advanced oxidation processes, their retention efficiencies for highly polar and recalcitrant PFASs are exceptionally low. Furthermore, the biotransformation of precursors during biological treatment often paradoxically exacerbates effluent concentrations, rendering STPs concentrated and continuous release points for PFASs into surface waters basin-wide. Finally, ubiquitous non-point source inputs constitute the environmental baseline that sustains the basin’s baseline concentrations. At locations such as the Yellow River estuary, as well as the tourist area (S1) and traditional agricultural zone (S8) investigated in this study, atmospheric deposition, routine consumption of fluorinated detergents and personal care products, alongside small-scale agricultural runoff, collectively maintain the baseline contamination levels in the macroscopic environment.

In summary, although PFOA and PFOS have been officially incorporated into China’s List of New Pollutants for Priority Management with strict prohibitions on their production and application, they will persist under the dual constraints of massive industrial structural inertia and their intrinsic extreme recalcitrance. Consequently, over a prolonged period in the future, these two substances will remain the core regulatory benchmarks for the targeted abatement of emerging contaminants and the interception of ecological risks within the Yellow River Basin.

### 3.3. Environmental Risk Assessment of PFOA and PFOS

#### 3.3.1. Ecological Risk Assessment

**Figure 3:**
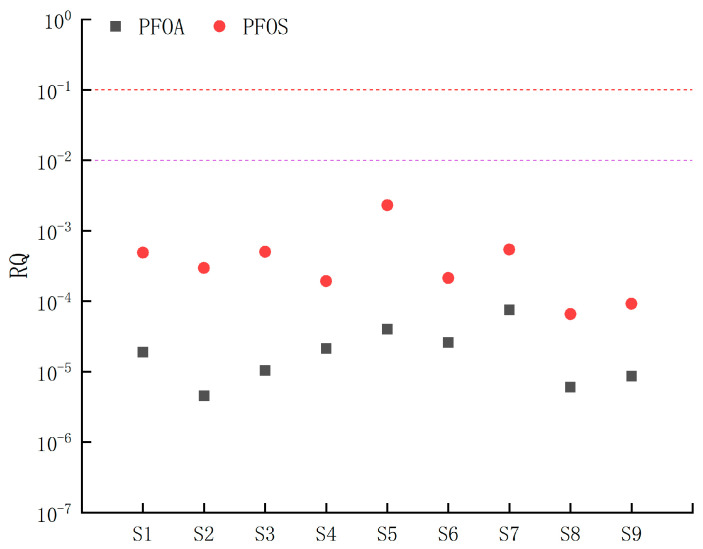
Ecological risk quotients (RQ) for PFOA and PFOS at different sampling sites in the surface water of the Yellow River (Henan Section).

To ensure a conservative and precautionary approach in the ecological risk assessment, this study adopted the predicted no-effect concentrations (PNECs) based on the most sensitive aquatic toxicological endpoints from existing literature (PNEC: 100 μg/L for PFOA; 1 μg/L for PFOS) [29,30] as quantitative benchmarks. The Risk Quotient (RQ) evaluation results (Figure 3) demonstrated that across all nine sampling cross-sections (S1–S9) in the Henan section of the Yellow River Basin, the RQ values for both PFOA and PFOS were far below the threshold of 0.01. This indicates that the current occurrence levels of the target pollutants pose a negligible direct ecological risk to the regional aquatic ecosystem.

Regarding the compositional differences, it is noteworthy that although the measured environmental concentrations of PFOS were generally lower than those of PFOA, the resultant RQ values for PFOS (10^−4^~10^−3^) were paradoxically an order of magnitude higher than those for PFOA (10^−5^~10^−4^). This discrepancy is attributed to its exceedingly low PNEC value, which reflects a higher biological toxicity sensitivity. Notably, this risk evaluation is confined to the aqueous exposure pathway. Given its pronounced hydrophobicity and high bioaccumulation potential, PFOS readily accumulates in sediments and biomagnifies across the food chain. Consequently, excluding benthic and dietary exposure routes from the current model implies that the holistic ecological risk may be underestimated. Spatially, the relatively high RQ values were primarily concentrated at cross-sections such as S5 (STP effluent) and S7 (river outfall), which perfectly aligns with the characteristics of intensive industrial point-source inputs identified in the preceding source apportionment. This paradoxical phenomenon of “low concentration occurrence yet high risk contribution” emphatically highlights that PFOS must be prioritized as a core target for prevention and targeted abatement within future environmental regulatory frameworks for emerging contaminants in the basin.

Further horizontal comparisons with other typical basins in China revealed that the overall ecological risk level of PFASs in the Henan section of the Yellow River was lower than that in the Dawen River, a downstream tributary profoundly impacted by high-load sewage discharges [9]. However, it was generally comparable to the risk levels observed in the surface water of the Shuangtaizi River [31] and typical STP-receiving rivers in northern China [32]. In conclusion, although the surface water in the study area has been universally subjected to baseline contamination by PFOA and PFOS, the current exposure loads largely remain within the controllable environmental carrying capacity of the water bodies and have not yet posed a substantive threat to the structural and functional stability of the regional aquatic ecosystem.

#### 3.3.2. Human Health Risk Assessment

Given the profound environmental persistence and bioaccumulation of PFASs, although the nine monitored cross-sections are not predominantly direct drinking water sources, they serve as critical reservoirs for agricultural irrigation and groundwater recharge along the basin. Therefore, conducting a health risk assessment via the drinking water ingestion pathway holds substantial prospective early-warning significance. In the absence of unified Health-Based Guidance Values (HBGVs) for PFASs in China, this study adopted the stringent Reference Dose (RfD: 20 ng/(kg·d)) established by the US EPA [33]. This standard has been widely applied in domestic PFAS aquatic environmental risk assessment with a mature evaluation framework, facilitating horizontal comparisons between domestic and international research findings [16,34]. Integrating the core exposure parameters extracted from the Report on the Nutrition and Chronic Disease Status of Chinese Residents [16] (Table 3), the Health Risk (HR) values via drinking water ingestion were systematically calculated for four specific demographics: adult males, adult females, male children, and female children.

**Figure 4:**
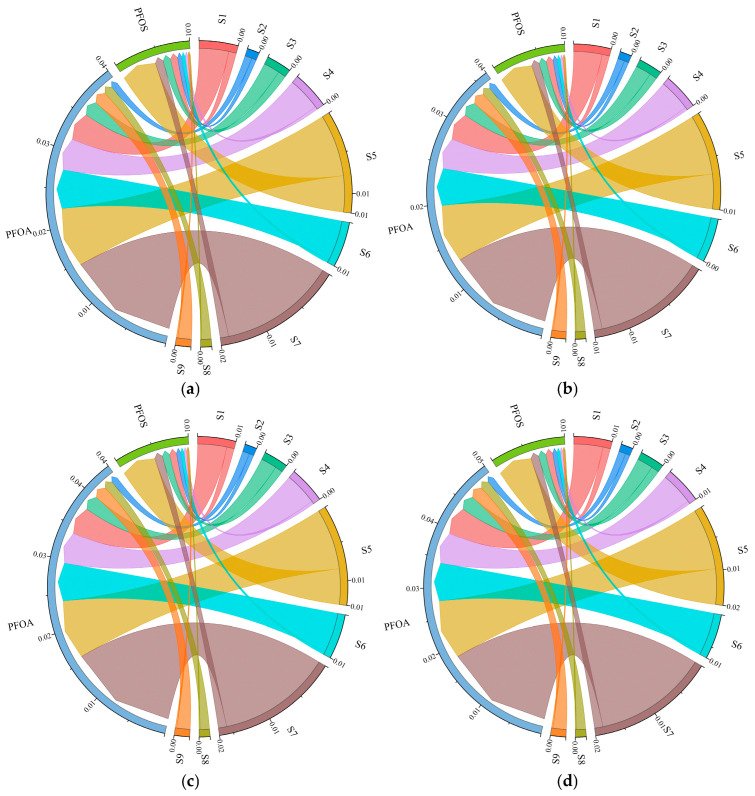
Health risk (HR) assessment of PFOA and PFOS via drinking water ingestion for four population subgroups at different sampling sites ((**a**): adult male, (**b**): adult female, (**c**): male child, (**d**): female child).

The multidimensional visual risk profiles (Figure 4) clearly elucidate the health exposure levels of the target pollutants, along with their spatial and demographic heterogeneities within the study area. First, regarding the baseline risk levels, the evaluation demonstrated that the HR values induced by PFOA and PFOS in the surface water of the Henan section were on the order of 10^−4^ to 10^−2^ for both adults and children. The HR values across all monitored cross-sections were far below the potential risk threshold of 0.1. This indicates that under the current water quality exposure levels, the probability of the target pollutants causing non-carcinogenic health impairments to surrounding residents is exceedingly low, implying that the health risks temporarily remain within a safe and controllable range.

Second, concerning spatial contributions and population sensitivity, the health risk hotspots were primarily concentrated at cross-sections S7 (downstream of the Jili District outfall) and S5 (effluent of the Mengzhou No. 2 STP). Although the PFOA concentration at site S7 exceeded the US EPA MCL of 4 ng/L, it should be noted that this standard applies to finished drinking water. When viewed as untreated surface water, this value serves primarily as a conservative benchmark indicating localized environmental pressure rather than an immediate human health threat, which is consistent with the calculated low HR values. This spatial pattern perfectly coincides with the previously identified ecological risk hotspots, further substantiating that high-load industrial discharges exert a decisive impact on the multidimensional environmental risks of receiving waters. Regarding demographic sensitivity, while the HRs across all age and gender groups consistently fell within the safe zone, a distinct age-dependent trend was observed. To avoid repetitive group-by-group descriptions, it is crucial to highlight that pediatric populations exhibited notably higher exposure loads compared to adults. This disproportionate health burden is primarily governed by children’s heightened environmental exposure vulnerability and intrinsic physiological sensitivity.

Behaviorally and physically, children possess a significantly higher daily water ingestion rate per unit of body weight, thereby amplifying their baseline intake of exogenous fluorinated chemicals. Toxicologically, children are in critical developmental windows. Their immature metabolic, immune, and xenobiotic-detoxification systems render them inherently more susceptible to the potential bioaccumulation and systemic disruptions caused by PFOA and PFOS. Consequently, pediatric cohorts must be prioritized as the most sensitive demographic targets in future long-term epidemiological and environmental biomonitoring across the basin.

Finally, from the perspective of horizontal basin comparisons and forward-looking early warnings, the mean assessment results of this study are generally consistent with those reported for the Wei River Basin [16] and the Beijiang River Basin [35]. Specifically, under current macroscopic exposure benchmarks, PFOA and PFOS are ubiquitous in surface waters but do not yet pose a direct public health threat. As the international toxicological community’s comprehension of PFAS health hazards deepens, global regulatory limits are exponentially tightening. For instance, in April 2024, the US EPA mandated an extraordinarily stringent Maximum Contaminant Level (MCL) of 4.0 ng/L for PFOA in drinking water. The peak PFOA concentration observed at cross-section S7 (7.46 ng/L) is relatively high when compared to the latest US EPA maximum contaminant levels for drinking water. However, considering the distinct designated uses of surface water bodies, this threshold serves primarily as a comparative reference. Nevertheless, given the overall ecological and health risk profile of the region, we recommend implementing long-term monitoring of emerging contaminants in primary discharge zones.

Although the calculated health risks based on RfDs remain low, it is crucial to recognize the global trend towards increasingly stringent toxicological benchmarks (e.g., the lowered RfDs for PFOA and PFOS recently issued by the US EPA). When re-evaluating our data against these latest international standards, the potential long-term human health risks at specific sites are significantly amplified, warranting heightened vigilance from regulatory authorities.

## 4. Conclusions

This study comprehensively investigated the occurrence, spatial evolution, and risk profiles of PFOA and PFOS across nine typical functional cross-sections in the Henan section of the Yellow River Basin. The primary conclusions are drawn as follows:

(1) The spatial distribution of these target pollutants exhibited ubiquitous exposure that was deeply coupled with the regional industrial layout. Specifically, with a 100% detection rate and PFOA maintaining absolute concentration dominance, the contamination levels displayed a distinct hierarchical decreasing gradient: industrial river outfalls > industrial sewage treatment plant (STP) effluents > densely populated urban areas > traditional agricultural zones and baseline tributaries.

(2) This pronounced spatial heterogeneity is fundamentally driven by intensive industrial point-source inputs. Source apportionment precisely identified the Jili District River outfall and the Mengzhou No. 2 STP as the most prominent pollution hotspots within the monitored network. Their high-load discharges primarily originate from the continuous effluent input of dense industrial clusters, including leather processing, fine chemicals, and advanced equipment manufacturing, thereby providing precise spatial coordinates for future targeted source-level control of emerging contaminants.

(3) Despite the continuous inputs from these industrial hotspots, multidimensional risk assessments confirmed that the overall environmental exposure currently remains within a controllable baseline. Both the ecological risks for aquatic organisms (RQ < 0.01) and the human health risks via drinking water ingestion (HR < 0.1) are maintained at a safe magnitude. It should be noted that the RfD adopted in this study is relatively conservative, leading to a somewhat optimistic health risk assessment; if more stringent contemporary health-based guidance values, such as those established by the European Food Safety Authority, were applied, the calculated risk metrics would elevate and potentially approach the safety thresholds. Nevertheless, two latent risk characteristics warrant attention. First, a toxicological paradox was observed: despite its exceedingly low environmental concentration, PFOS overwhelmingly dominates the ecological risk contribution due to its disproportionately high biological toxicity. Second, driven by higher body weight-normalized water ingestion rates and heightened physiological sensitivity (e.g., developing metabolic and detoxification systems), children exhibited notably higher exposure burdens than adults. This underscores that pediatric demographics must be prioritized as a critical defense line in the long-term biomonitoring of the basin.

(4) Furthermore, from a forward-looking regulatory perspective, the findings sound a critical early warning. Although the monitored concentrations currently comply with China’s existing water quality redlines, the PFOA loads at key discharge terminals (peaking at 7.46 ng/L downstream of the S7 outfall) have significantly breached the extraordinarily stringent Maximum Contaminant Level (MCL: 4.0 ng/L) newly promulgated by the US EPA in April 2024. Confronted with the highly recalcitrant nature of PFASs and globally tightening regulatory frameworks, it is imperative for basin management authorities to implement a dynamic, lifecycle-based early-warning network. This paradigm shift will compel high-risk fluorochemical-related industries to initiate advanced water purification upgrades, thereby solidifying the foundational water ecological barrier for the major national strategy of the Yellow River Basin.

In summary, while this study provides a crucial baseline ‘snapshot,’ the dynamic environmental fate of PFAS is inevitably governed by the unique hydrological rhythms of the Yellow River Basin. Seasonal fluctuations manifest as precipitation-driven dilution effects during the wet season and severe concentration effects during the dry season, both of which profoundly modulate localized exposure risks. To transcend current mechanistic limitations, future research should transition toward a holistic, multi-compartment framework. Chemically, it is imperative to include the screening of polyfluoroalkyl precursors because they act as substantial hidden sources that continuously degrade into terminal perfluoroalkyl acids. Ecologically, the risk assessment must expand beyond the aqueous phase to evaluate benthic organism exposure and biomagnification along the food web. Ultimately, integrating these multi-media investigations with long-term seasonal dynamics and concurrent environmental metadata (e.g., pH, dissolved organic carbon, conductivity, and real-time hydrological flow) will rigorously elucidate partitioning behaviors, capture time-variant risk extremes, and guide adaptive environmental governance at the basin scale.

## Figures and Tables

**Table 1:** Sampling site information and functional positioning in the Yellow River Basin (Henan Section).

Sampling Site ID	Cross-Section Name	Functional Positioning	Characteristics of Major Pollution Sources
S1	Qingtian River	Drinking water source/Tourist area	Non-point sources from typical human activities (tourism, domestic sewage)
S2/S3	Mang River	Administrative assessment/Baseline	Comprehensive tributary input, reflecting the baseline level of the basin
S4/S5	STP Effluent	Industrial/Domestic receiving water	Point sources including paper, equipment manufacturing, leather, and biochemicals
S6	Upstream of outfall	Control cross-section	Baseline prior to the outfall discharge into the Yellow River
S7	Downstream of outfall	Key point-source impact zone	Direct impact from the outfall discharge in Jili District
S8	Panxi River	Agricultural activity zone	Non-point sources from typical traditional farmland cultivation
S9	STP Effluent	Chemical park receiving water	High-tech industrial point sources (e.g., nanomaterials, fine chemicals)

**Table 2:** Comparison of the occurrence characteristics and primary sources of typical PFASs in surface water across different sections of the Yellow River Basin.

Basin Section	Representative Sites	Predominant PFASs	Concentration Range of PFOA/PFOS (ng/L)	Primary Sources	Ref.
Upstream tributaries	Huangshui River	PFOA, PFOS, PFBA	3015.96/826.4	Textile processing (carpet manufacturing), STP effluents	[24]
	Dahei River	PFOA, PFHxA, PFOS	2.44/1.05	Fluorochemical industry, aqueous film-forming foams (AFFF)	[25]
Midstream tributaries	Fenhe River	PFOA, PFOS	2.49–4.79/3.54–16.23	Municipal domestic sewage, adjacent heavy industry wastewater	[26]
This study	Henan section of the Yellow River	PFOA, PFOS	0.45–7.46/0.08–2.32	Fine chemicals, leather manufacturing, outfall discharges	This study
Downstream mainstream & tributaries	Beiluo River/Qingjian River	PFOA, PFOS, FOSA	Mean: 31.6–165	Petroleum exploration and extraction activities	[27]
	Dongying section	PFOA, PFBA	2.04–2.68/0.86–1.31	Atmospheric deposition, domestic sewage	[28]

**Table 3:** Average body weight and daily drinking water ingestion parameters for adults and children.

Subject	Gender	BW (kg)	IR (L/d)
Adult	Male	66.2	2.591
	Female	57.3	1.994
Child	Male	20.8	0.877
	Female	20.2	0.986

Note: BW = Body weight; IR = Ingestion rate.

## Data Availability

The original contributions presented in this study are included in the article. Further inquiries can be directed to the corresponding authors.
